# Dissecting the Phenotypic Regulation Characteristics of Lodging Resistance in Dry Direct Seeding Rice: Insights from Stem Mechanics and Structural Traits

**DOI:** 10.3390/plants15091287

**Published:** 2026-04-22

**Authors:** Zhiqiang Tang, Chao Liang, Li Wen, Wurina Sun, Jicong Liu, Zuobin Ma, Wenjing Zheng, Shu Wang, Hui Wang

**Affiliations:** 1 Seed Industry Innovation Research Institute, Liaoning Academy of Agricultural Sciences, Shenyang 110101, China; 2 Inner Mongolia Key Laboratory of Rice Breeding Innovation in Northern Cold Regions, Hinggan League Academy of Agricultural and Animal Husbandry Sciences, Ulanhot 137400, China; 3 Rice Research Institute of Liaoning Province, Liaoning Academy of Agricultural Sciences, Shenyang 110101, China; 4 College of Agronomy, Shenyang Agricultural University, Shenyang 110866, China

**Keywords:** dry direct seeding rice, lodging resistance, variety, stem mechanics

## Abstract

Lodging is a major constraint limiting grain yield in dry direct seeding rice (DDSR), yet the key traits and phenotypic relationships governing lodging resistance in *japonica* varieties adapted to this system remain poorly understood. This study evaluated 79 *japonica* accessions over two years in Shenyang, Northeast China, to dissect phenotypic variation in lodging index and identify ideotypes for breeding. Based on hierarchical clustering, varieties were classified into strong lodging resistance (SLR), medium lodging resistance (MLR), and weak lodging resistance (WLR) types, with SLR varieties achieving lodging indices 27.4–31.8% lower than those of MLR and 63.2–83.8% lower than those of WLR varieties. SLR varieties reduced lodging risk by coordinately balancing whole-plant bending moment and stem breaking resistance: plant height and center-of-gravity height were 5.2–10.7% lower, while basal internode bending stress was 27.9–81.9% higher than in other types. Structural equation modeling identified culm dry weight, inner diameter, and culm phenotype index as primary determinants of lodging variation. Notably, despite 11.0–13.7% fewer spikelets per panicle, SLR varieties maintained grain yields comparable to those of WLR varieties through compensatory increases in grain-filling rate (6.7–7.3%) and 1000-grain weight (8.1–8.7%). These findings demonstrate that optimizing basal internode structure and enhancing culm tissue density can simultaneously improve lodging resistance and preserve yield potential, providing a practical framework for breeding lodging-resistant, high-yielding *japonica* varieties for DDSR systems in Northeast China.

## 1. Introduction

Rice (*Oryza sativa* L.) is a staple food for more than half of the global population and is widely cultivated in many countries [1]. However, water scarcity, labor shortages, and the challenges posed by climate change are placing significant constraints on the sustainability of rice production worldwide [2,3]. In this context, direct seeding rice (DSR) has emerged as a critical agricultural practice with the potential to address these issues effectively [4]. Compared with conventional transplanted rice, DSR eliminates the labor-intensive processes of nursery raising and seedling transplanting, thereby substantially reducing production costs and labor requirements [5]. Moreover, DSR requires less water input during the early growth stages and facilitates mechanized cultivation, making it particularly suitable for regions facing increasing water scarcity and rural labor migration [6]. Consequently, this cultivation method has increasingly become the preferred choice for rice production in numerous developing countries [5].

Despite its advantages, the large-scale cultivation of direct seeding rice introduces specific challenges, including susceptibility to lodging, uncontrolled weeds and poor crop establishment [6,7,8]. Among these constraints, lodging is one of the most critical factors restricting the yield potential of direct seeding rice. Extensive lodging destroys the normal canopy structure of crops, thereby inhibiting photosynthesis and carbohydrate transport. In direct seeding systems, yield losses due to lodging have been reported to range from 2% to 35%, with losses as high as 0.7–2.0 t ha^−1^ [9,10]. The resulting physiological disruption also leads to deterioration in grain quality and reduced efficiency of mechanical harvesting [11,12].

Understanding the mechanisms underlying lodging susceptibility in DSR requires a detailed examination of the biomechanical factors governing stem failure. Lodging refers to the permanent displacement of plants from their upright position, occurring when inherent mechanical parameters prove insufficient to withstand adverse environmental conditions [13]. It is generally classified into two distinct types: root lodging and stem lodging. The former results from the failure of the root–soil anchoring system or root displacement within the soil [14], whereas the latter involves the tilting or horizontal displacement of the plant due to structural failure of the culm [15]. Notably, the lodging risk profile of dry direct seeding rice (DDSR) differs fundamentally from that of conventional transplanted rice. In the transplanting system, seedlings develop a robust, deep-penetrating root system due to the nursery stage and transplanting shock, whereas DDSR plants are characterized by a shallower root distribution concentrated in the topsoil layer, which weakens the root–soil anchorage and increases susceptibility to root lodging [16]. Furthermore, the high seeding densities typically employed in DDSR to ensure adequate stand establishment exacerbate intra-specific competition, leading to etiolated and slender basal culms with reduced mechanical strength [17]. This structural weakness is particularly pronounced in Northeast China, where *japonica* rice varieties bred primarily for the traditional transplanting system are now increasingly adapted to dry direct seeding. Although direct-seeded rice is generally considered more susceptible to root lodging than transplanted rice due to its shallower root distribution, recent evidence indicates that stem lodging is actually more prevalent in DDSR systems. This type of lodging occurs primarily at the basal second internode as a consequence of high seeding rates, which reduce the breaking resistance of rice internodes [18,19,20].

The breeding of lodging-resistant varieties represents an important strategy for reducing lodging risk in direct seeding rice [21]. The variation in lodging resistance among rice varieties is closely associated with both the morphological architecture and mechanical properties of the stem [22]. It is widely recognized that plant height plays a crucial role in lodging susceptibility, with tall-statured, large-panicle varieties generally exhibiting higher lodging risk [16,23,24]. A suitable plant height combined with an optimized internode configuration, specifically shortened basal internodes and elongated upper internodes below the panicle, has been proposed as an effective strategy for improving lodging resistance [17,20]. Nevertheless, plant height alone does not fully account for the observed variation in lodging resistance among genotypes. Evidence suggests that stem strength, determined by intrinsic culm properties, such as wall thickness, tissue density, and mechanical tissue development, may exert an equally important influence [25]. Multiple strategies have been employed to enhance stem strength and improve lodging resistance in rice. For instance, positional cloning technology has been successfully utilized to identify and introgress genes associated with increased stem diameter, thereby enhancing the lodging resistance of otherwise high-stalk varieties [26,27,28,29]. Alternatively, increasing culm wall thickness represents another effective approach, as thicker walls substantially enlarge the cross-sectional area of the basal internode. Indeed, previous research has demonstrated that this structural modification can double the breaking strength of the basal internode [30]. Beyond geometric modifications, improving the intrinsic density of culm tissues and the surrounding leaf sheath offers an additional avenue for strengthening stem mechanical properties. Greater culm density directly enhances the material strength of the stem, while higher leaf sheath density provides supplementary physical support to the enclosed culm, particularly during the grain-filling stage when lodging risk is greatest [31,32].

Despite extensive research on lodging resistance in transplanted rice, systematic understanding of DDSR remains insufficient [20,33,34]. Existing studies on DDSR lodging resistance have primarily focused on isolated morphological traits or single mechanical parameters, and the few investigations conducted under direct seeding conditions have typically employed a limited number of varieties [17,19,35]. Critically, the specific phenotypic relationships governing the interplay between plant architecture, internode structure, and whole-plant mechanical indicators in DDSR remain poorly defined. Furthermore, the trade-off between enhanced lodging resistance and grain yield potential in *japonica* varieties adapted to dry direct seeding in Northeast China remains largely unexplored. Consequently, a comprehensive evaluation that simultaneously examines morphological architecture, stem biomechanics, and yield performance across diverse germplasm is urgently needed to inform breeding strategies for DDSR.

Therefore, a two-year field experiment was carried out with 79 *japonica* inbred rice varieties popularly utilized in Northeast China. The objectives of the present study were: (1) to evaluate the variation in morphological characteristics, mechanical parameters, and basal internode traits among different varieties; (2) to determine the impact of differential lodging resistance on yield performance and elucidate the underlying trade-off or synergy; and (3) to identify the phenotypic relationships and causal pathways through which key morphological and mechanical traits, including lodging index, plant height component index, and culm structural properties, modulate lodging resistance under dry direct seeding conditions. The results of this study are expected to provide valuable guidelines for breeders aiming to develop new varieties with high lodging resistance specifically suited to direct seeding rice systems.

## 2. Results

### 2.1. Lodging Index and Related Mechanical Parameters

**Figure 1:**
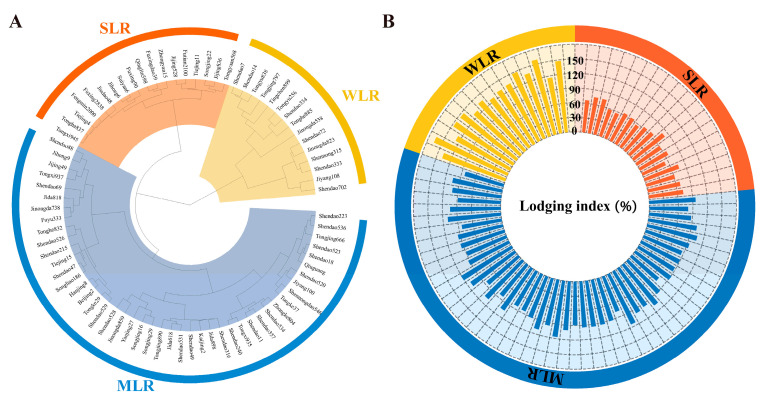
Hierarchical clustering (**A**) of 79 dry direct seeding rice varieties and lodging indices (**B**) of different variety types. SLR, strong lodging resistance varieties; MLR, medium lodging resistance varieties; WLR, weak lodging resistance varieties.

The lodging index (LI) of 79 tested varieties ranged from 51.7 to 234.0% and from 42.6 to 176.4% in 2020 and 2021, respectively. Ward’s method was used for hierarchical cluster analysis, and 79 varieties were divided into different categories using the calculated lodging index of each variety in 2020 and 2021. The tested rice varieties were separated into three variety types, including strong lodging resistance (SLR), medium lodging resistance (MLR), and weak lodging resistance (WLR), using Ward’s method for hierarchical clustering (Figure 1). Compared with MLR and WLR, the LI of SLR decreased by 27.4% and 83.8% and by 31.8% and 63.2% in 2020 and 2021, respectively. ANOVA showed that there was no significant difference in lodging index among years; in contrast, the variety type and the interaction between variety type and year significantly influenced lodging index (Table 1).

**Figure 2:**
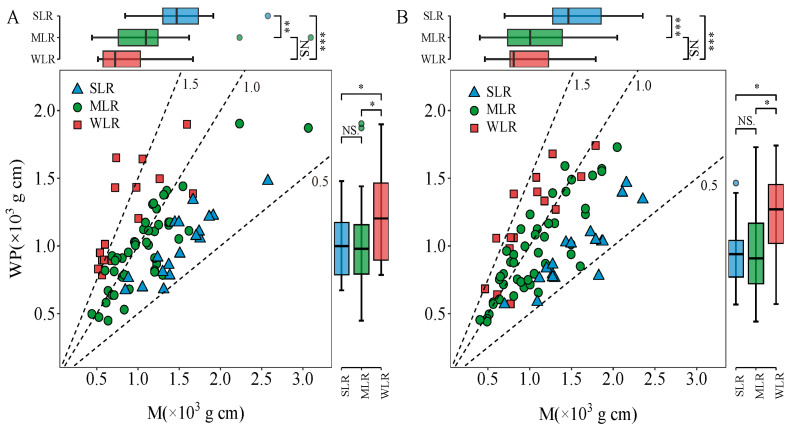
The relationship between bending moment of the whole plant (WP) and bending moment at breaking (M) (**A**,**B**) in the 2020 and 2021 growing seasons, respectively. Dotted lines show the lodging index (LI). SLR, strong lodging resistance varieties; MLR, medium lodging resistance varieties; WLR, weak lodging resistance varieties. The boxes within each plot represent the interquartile ranges, with the means marked by horizontal lines; the whiskers indicate the minimum and maximum values. NS., not significant at *p* = 0.05; *, *p* < 0.05; **, *p* < 0.01; ***, *p* < 0.001.

The LI could be divided into two parts, including the bending moment of the whole plant (WP) and the bending moment at breaking (M). The year and variety type had a significant effect on WP (Table 1). Additionally, the interaction between these factors also showed a significant effect on WP. The values for WP in 2020 displayed the following trend among variety types: MLR < SLR < WLR (Figure 2A), while in 2021, the WP values of different variety types were SLR < MLR < WLR (Figure 2B). Compared to the WLR, the SLR and MLR decreased the average value of WP in 2020 and 2021 by 18.8% and 19.5% and by 20.0% and 19.1%, respectively. M was not significantly influenced by the year and the interaction between year and variety type. The variation in M in both years displayed the following trend: SLR > MLR > WLR (Figure 2A,B). The lodging resistance of varieties increased significantly with the increase in M. The average value of SLR in M (for both years) was 38.0% and 72.4% higher than that of MLR and WLR, respectively.

**Figure 3:**
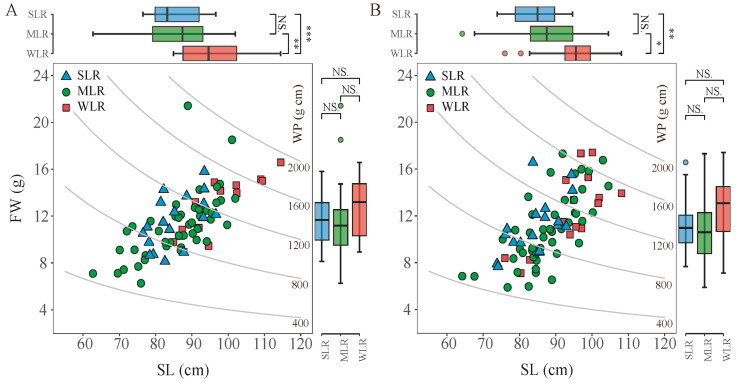
The relationship between the fresh weight of the plant part above the broken point (FW) and the length from the broken point to the panicle top (SL) (**A**,**B**) in the 2020 and 2021 growing seasons, respectively. Curved lines show the bending moment of the whole plant (WP). SLR, strong lodging resistance varieties; MLR, medium lodging resistance varieties; WLR, weak lodging resistance varieties. The boxes within each plot represent the interquartile ranges, with the means marked by horizontal lines; the whiskers indicate the minimum and maximum values. NS., not significant at *p* = 0.05; *, *p* < 0.05; **, *p* < 0.01; ***, *p* < 0.001.

As shown in Figure 3, the WP value is determined by the length from the broken point to the panicle top (SL) and the fresh weight of the plant part above the broken point (FW). In this study, the SL values of varieties were consistent over the two years, ranking from lowest to highest as SLR < MLR < WLR. Compared to WLR, the mean SL values of SLR and MLR were reduced significantly by 10.9% and 8.6%, respectively. During the two years, the FW values followed the order WLR > SLR > MLR. Specifically, the FW values of SLR and MLR were lower than that of WLR, showing reductions of 8.8% and 12.0%, respectively.

**Figure 4:**
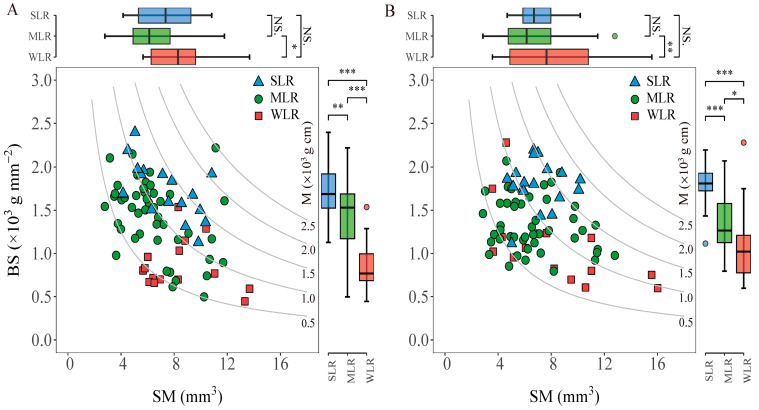
The relationship between bending stress (BS) and cross-section modulus (SM) (**A**,**B**) in the 2020 and 2021 growing seasons, respectively. Curved lines show the breaking moment (M). SLR, strong lodging resistance varieties; MLR, medium lodging resistance varieties; WLR, weak lodging resistance varieties. The boxes within each plot represent the interquartile ranges, with the means marked by horizontal lines; the whiskers indicate the minimum and maximum values. NS., not significant at *p* = 0.05; *, *p* < 0.05; **, *p* < 0.01; ***, *p* < 0.001.

M is determined by the cross-section modulus (SM) and bending stress (BS). The analysis of variance showed that the variety type had a significant effect on both SM and BS (Table 1). Among variety types, the WLR variety type consistently exhibited the highest SM values, averaged across the two years. In contrast, the lowest SM values were found in the MLR variety type (Figure 4). Compared to WLR, the SM value of MLR significantly decreased by 19.1% in 2020 and 22.9% in 2021, whereas no significant difference was observed between SLR and WLR. The BS was significantly affected by the physical characteristics of the stem. In this study, the differences in the BS values among different variety types were significant, showing a trend of SLR > MLR > WLR. Compared with MLR and WLR, the average BS value of SLR in the two growing seasons increased by 21.3% and 101.8% and by 34.9% and 39.8%, respectively.

### 2.2. Morphological Traits of Individual Plants

**Figure 5:**
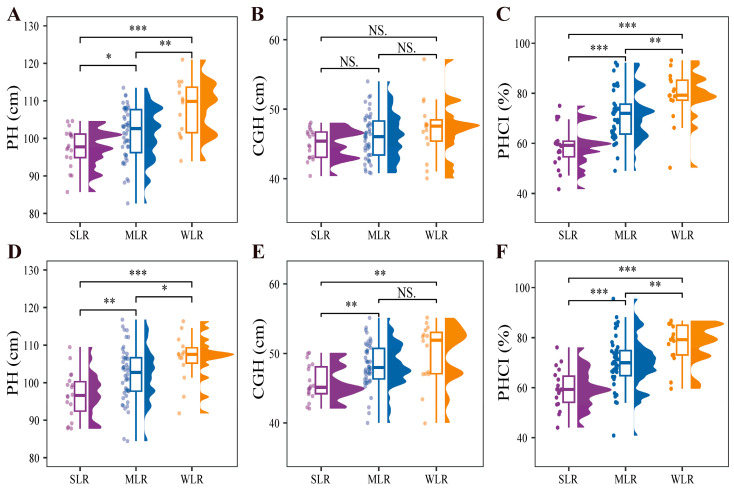
Raincloud plots for PH (**A**,**D**), CGH (**B**,**E**), and PHCI (**C**,**F**) of different variety types in the 2020 and 2021 growing seasons, respectively. PH, plant height; CGH, center-of-gravity height; PHCI, plant height component index of basal 2nd internode; SLR, strong lodging resistance varieties; MLR, medium lodging resistance varieties; WLR, weak lodging resistance varieties. The boxes within each plot represent the interquartile ranges, with the means marked by horizontal lines; the whiskers indicate the minimum and maximum values. Scatter plots to the left and density plots to the right show the individual values and distribution of values, respectively. NS., not significant at *p* = 0.05; *, *p* < 0.05; **, *p* < 0.01; ***, *p* < 0.001.

Plant height was significantly affected by variety type (Table 2), with SLR varieties consistently exhibiting the lowest values across both years (Figure 5A,D). SLR varieties benefit from reduced plant height, which shortens the lever arm from the basal internode to the panicle and thereby directly decreases the bending moment exerted by the upper plant mass on the basal internode. Consistent with this, the center-of-gravity height (CGH) was also lower in the SLR varieties, with significant reductions of 5.2% and 8.3% relative to MLR and WLR, respectively, in 2021, though no significant differences were observed in 2020 (Figure 5B,E), further reducing the effective lever arm through which gravitational forces act on the stem and diminishing the whole-plant bending moment. The plant height component index (PHCI) also differed markedly among variety types. With an increase in the lodging resistance of the varieties, there was an observed decreasing trend in the plant height component index (Figure 5C,F). Comparatively, the plant height component index of the SLR and MLR reduced by 24.3% and 9.6%, respectively, in contrast to the WLR. Collectively, these morphological adjustments in SLR varieties enhance lodging resistance by synergistically reducing the whole-plant bending moment.

### 2.3. Internode Traits

**Figure 6:**
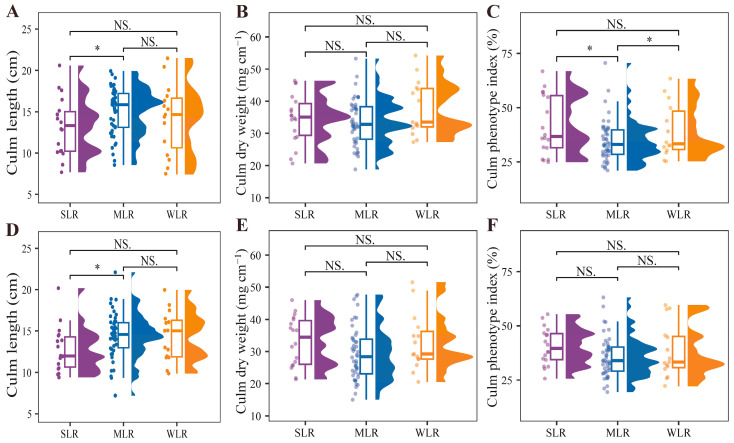
Raincloud plots for culm length (**A**,**D**), culm dry weight (**B**,**E**) and culm phenotype index (**C**,**F**) of different variety types in the 2020 and 2021 growing seasons, respectively. SLR, strong lodging resistance varieties; MLR, medium lodging resistance varieties; WLR, weak lodging resistance varieties. The boxes within each plot represent the interquartile ranges, with the means marked by horizontal lines; the whiskers indicate the minimum and maximum values. Scatter plots to the left and density plots to the right show the individual values and distribution of values, respectively. NS., not significant at *p* = 0.05; *, *p* < 0.05.

The culm length of the basal second internode differed significantly among variety types (Table 3), with SLR varieties consistently exhibiting the shortest internodes across both years (Figure 6A,D). Specifically, the basal internode length of the SLR varieties was reduced by 11.9% and 11.1% compared with the MLR varieties in 2020 and 2021, respectively. This reduction in basal internode length lowers the plant’s center of gravity and shortens the lever arm through which wind and gravitational forces act on the stem base, thereby decreasing the whole-plant bending moment and enhancing lodging resistance. Culm dry weight and culm phenotype index (CPI) were significantly affected by the interaction between year and variety type (Table 3). In 2020, both traits followed the order MLR < SLR < WLR, whereas in 2021, the ranking shifted to MLR < WLR < SLR (Figure 6B,C,E,F). Notably, the CPI of SLR varieties exceeded that of MLR varieties by 20.2% in 2020. The elevated CPI reflects a more robust basal internode architecture, characterized by a greater diameter relative to the internode length. Collectively, this compact and dense stem morphology directly enhances lodging resistance by increasing the bending stress of the basal internode.

**Figure 7:**
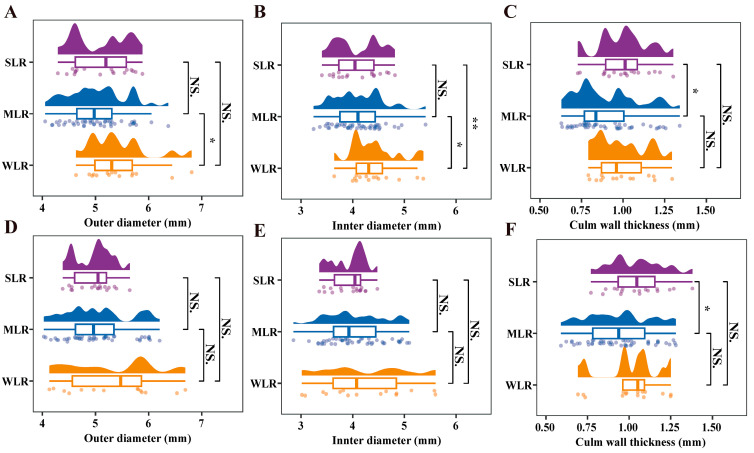
Raincloud plots for outer diameter (**A**,**D**), inner diameter (**B**,**E**) and culm wall thickness (**C**,**F**) of different variety types in the 2020 and 2021 growing seasons, respectively. SLR, strong lodging resistance varieties; MLR, medium lodging resistance varieties; WLR, weak lodging resistance varieties. The boxes within each plot represent the interquartile ranges, with the means marked by horizontal lines; the whiskers indicate the minimum and maximum values. Scatter plots to the left and density plots to the right show the individual values and distribution of values, respectively. NS., not significant at *p* = 0.05; *, *p* < 0.05; **, *p* < 0.01.

The outer diameter was significantly influenced by variety type and the interaction between variety type and year (Table 3). In 2020, the outer diameter of the MLR varieties was 7.9% smaller than that of the WLR varieties, while no significant differences were observed among variety types in 2021 (Figure 7A,D). Inner diameter and culm wall thickness were both significantly affected by variety type (Table 3). In 2020, the inner diameters of the SLR and MLR varieties were 7.6% and 7.7% smaller than that of the WLR varieties, respectively (Figure 7B,E). Furthermore, the culm wall thickness of SLR was 12.5% and 11.1% greater than that of MLR in 2020 and 2021, respectively (Figure 7C,F). A smaller inner diameter, when combined with adequate culm wall thickness, indicates a greater proportion of load-bearing tissue within the cross-sectional area. The combination of reduced inner diameter and increased culm wall thickness in SLR varieties optimizes the trade-off between structural economy and mechanical strength, thereby conferring superior lodging resistance under dry direct seeding conditions.

### 2.4. Grain Yield, Yield Components and Panicle Traits

The grain yield of the tested varieties ranged from 4.31 t ha^−1^ to 10.97 t ha^−1^ and from 4.48 t ha^−1^ to 11.03 t ha^−1^ in 2020 and 2021, respectively. Despite substantial variation in lodging resistance among variety types, grain yield did not differ significantly among SLR, MLR, and WLR varieties in either year (Table 4). This demonstrates that the morphological and structural modifications conferring superior lodging resistance in SLR varieties do not inherently constrain yield potential. Analysis of yield components revealed significant differences among variety types in spikelets per panicle, spikelets per unit area, grain-filling rate, and 1000-grain weight (Table 4). SLR varieties consistently produced fewer spikelets per panicle than WLR varieties, with reductions of 13.7% in 2020 and 11.0% in 2021 and corresponding decreases in spikelets per unit area of 16.2% and 13.8%, respectively. However, these reductions in sink size were fully offset by significant improvements in sink strength and grain development. Specifically, SLR varieties achieved grain-filling rates that were 6.7% and 7.3% higher than those of WLR varieties in 2020 and 2021, respectively, and 1000-grain weights that were 8.1% and 8.7% greater. This compensatory pattern indicates that when one yield component is constrained, others increase correspondingly to offset the negative impact, provided that source–sink supply capacity remains non-limiting.

**Figure 8:**
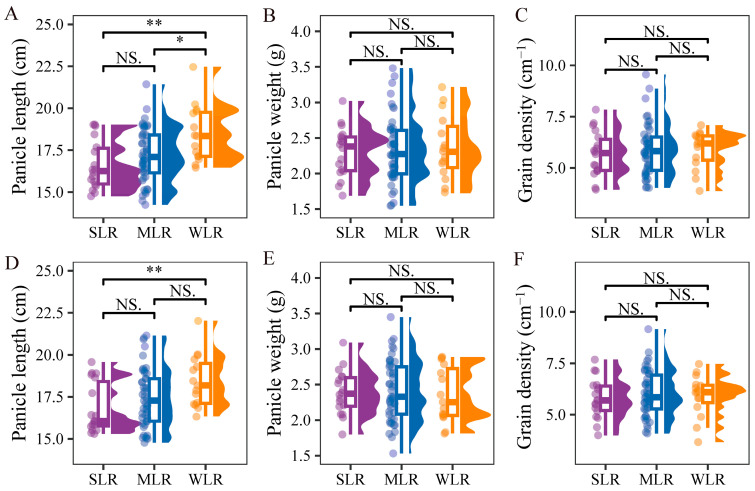
Raincloud plots for panicle length (**A**,**D**), panicle weight (**B**,**E**) and grain density (**C,F**) of different variety types in the 2020 and 2021 growing seasons, respectively. SLR, strong lodging resistance varieties; MLR, medium lodging resistance varieties; WLR, weak lodging resistance varieties. The boxes within each plot represent the interquartile ranges, with the means marked by horizontal lines; the whiskers indicate the minimum and maximum values. Scatter plots to the left and density plots to the right show the individual values and distribution of values, respectively. NS., not significant at *p* = 0.05; *, *p* < 0.05; **, *p* < 0.01.

Panicle length was significantly shorter in SLR varieties, with reductions of 9.5% averaged across both years, whereas panicle weight and grain density did not differ significantly among variety types (Figure 8). The observation that panicle weight remained unchanged despite shorter panicle length indicates that SLR varieties possess a more compact panicle architecture with higher grain packing density. This compact panicle morphology contributes to a lower center of gravity, thereby reducing lodging risk, while maintaining yield potential through efficient spikelet arrangement.

### 2.5. Correlation Analysis of Yield Traits and Lodging-Related Traits

**Figure 9:**
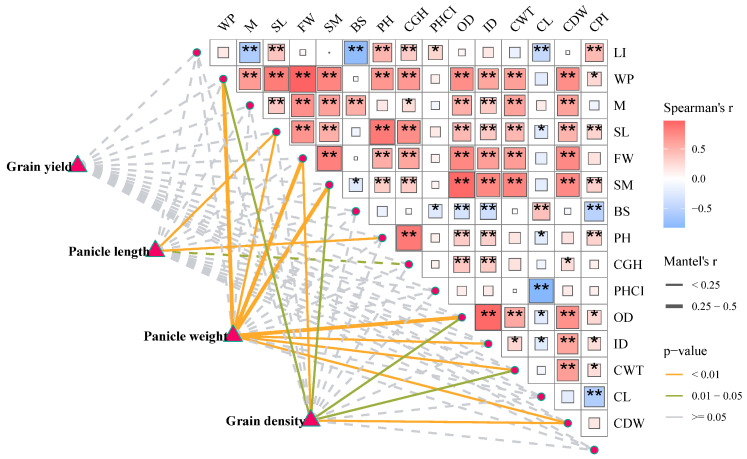
Spearman correlation and Mantel test analysis of yield-related traits and lodging-related traits, including lodging index (LI), bending moment of the whole plant (WP), bending moment at breaking (M), length from broken point to panicle top (SL), fresh weight of the plant part above the broken point (FW), cross-section modulus (SM), bending stress (BS), plant height (PH), center-of-gravity height (CGH), plant height component index (PHCI), outer diameter (OD), inner diameter (ID), culm wall thickness (CWT), culm length (CL), culm dry weight (CDW), and culm phenotype index (CPI). The upper triangular matrix in Figure 4 displays the Spearman correlation coefficients between the lodging-related traits, with colors representing the strength and direction of the correlations (blue for negative, red for positive). The size of the squares is proportional to the magnitude of the correlation coefficients, *, *p* < 0.05; **, *p* < 0.01. Overlaying the correlation matrix, lines represent significant relationships identified by the Mantel test, where line color indicates the *p*-value category (<0.001, 0.001–0.01, 0.01–0.05, ≥0.05), line thickness represents the Mantel’s r value (<0.25, 0.25–0.5, ≥0.5), and line type (solid or dashed) corresponds to the *p*-value significance levels.

Correlation matrix analysis (Figure 9) revealed distinct association patterns between lodging-related and yield-related traits. Lodging index (LI) exhibited strong negative correlations with bending moment at breaking (M, r = −0.58, *p* < 0.01) and bending stress (BS, r = −0.76, *p* < 0.01), reflecting the biomechanical principle that greater stem strength directly diminishes lodging susceptibility. Conversely, LI showed moderate to strong positive correlations with plant height (PH, r = 0.50, *p* < 0.01), center-of-gravity height (CGH, r = 0.35, *p* < 0.01), and length from broken point to panicle top (SL, r = 0.39, *p* < 0.01), confirming that taller plants with higher centers of gravity experience greater whole-plant bending moments. Among morphological traits, PH and CGH correlated positively with whole-plant bending moment (WP, r = 0.67 and 0.67, *p* < 0.01) and SL (r = 0.85 and 0.73, *p* < 0.01). The strong PH-SL correlation (r = 0.85) indicates that taller varieties possess longer lever arms, mechanically amplifying the basal bending moment. Plant height component index (PHCI) correlated negatively with BS (r = −0.25, *p* < 0.05), suggesting that a higher proportion of basal internodes reduces tissue-level material strength. This may reflect etiolation effects, where rapid internode elongation dilutes cell wall constituents and reduces vascular bundle density per unit length. Regarding yield associations, PH and SL showed moderate positive correlations with panicle length (r = 0.17–0.19, *p* < 0.05), indicating that taller varieties tend to produce longer panicles, which benefits yield potential but simultaneously elevates lodging risk. Stem structural traits (OD, CWT, and CDW) exhibited weak to moderate positive correlations with panicle weight (r = 0.21–0.25, *p* < 0.01) and grain density (r = 0.08–0.19, *p* < 0.05), suggesting that varieties with inherently stronger stems can support heavier panicles without disproportionate increases in lodging susceptibility.

### 2.6. Analysis of Factors Driving Variability in Lodging Index

**Figure 10:**
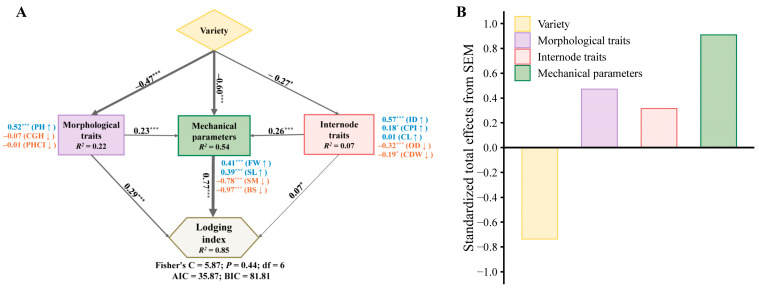
Structural equation models (SEMs) describing the direct and indirect effects of variety type, morphological traits, internode traits and mechanical parameters on lodging index (**A**). Total effects of relevant parameters on lodging index (**B**). The morphological traits, internode traits and mechanical parameters were divided into composite variables. Numbers adjacent to measured variables are their coefficients with composite variables. Numbers adjacent to arrows indicate the standardized effect size of the relationship. The thickness of the arrows represents the strength of the relationships. The significance levels for each predictor are as follows: * *p* < 0.05, *** *p* < 0.001. *R*^2^ denotes the proportion of variance explained for mechanical parameters and lodging index. Values of Fisher’s C with *P* in the models were >0.05, representing acceptable goodness of fit for the constructed model.

To further reveal the key factors driving the variability in lodging index, piecewise structural equation modelling (piecewiseSEM) was employed to evaluate the direct and indirect links between lodging index and related parameters (Figure 10A). The differences among varieties in morphological traits, internode characteristics, and mechanical parameters collectively explained the wide variation in the lodging index. Meanwhile, morphological traits and internode characteristics had a significant indirect effect on the lodging index by influencing mechanical parameters. Among the morphological traits, plant height was identified as the most important predictor driving the variation in mechanical parameters and the lodging index. In contrast, culm dry weight, inner diameter, and culm phenotype index played critical roles in enhancing stem strength. Furthermore, compared to morphological traits and internode traits, variety and mechanical parameters exhibited a greater total effect on the lodging index (Figure 10B).

## 3. Discussion

### 3.1. Differences in Lodging Resistance of Rice Germplasm Under Dry Direct Seeding

Lodging index is generally considered an indicator to evaluate the lodging resistance against stem breaking in rice varieties, with higher lodging index values indicating higher lodging risk [36,37,38]. In this study, the lodging indices of the tested varieties ranged from 42.6% to 234.0% and then were divided into three variety types based on hierarchical clustering, namely, SLR, MLR, and WLR (Figure 1). The wide range of lodging index values observed underscores the substantial genetic diversity in stem mechanical properties among Northeast China *japonica* germplasm, providing a rich resource for breeding selection. This magnitude of variation is comparable to, if not greater than, that reported in previous studies on transplanted rice populations [20,33], suggesting that the dry direct seeding environment may amplify phenotypic differences in lodging susceptibility among genotypes.

The variation in lodging index is determined by both the bending moment at breaking (M) and the bending moment of the whole plant (WP). Previous studies reported that the lodging index of inbred and hybrid varieties grown under transplanting conditions is more closely related to the breaking resistance than the bending moment [30,33]. However, it was reported that variations in the lodging index in direct seeding rice systems were attributed to bending moment rather than breaking resistance [19]. These seemingly contradictory findings reflect the complex genotype–environment–management interactions that govern lodging risk. In transplanted systems, where plants are spaced more widely and develop robust root systems, stem strength (M) becomes the primary limiting factor. Conversely, in high-density direct seeding systems, the etiolation of lower internodes due to mutual shading may shift the balance toward whole-plant architectural parameters (WP) as the dominant determinants [17,19]. In this study, SLR exhibited a significantly higher bending moment at breaking compared to the other variety types (Figure 2). Moreover, SLR also markedly reduced the bending moment of the whole plant relative to WLR. Therefore, SLR achieved a significant reduction in lodging index by effectively coordinating the bending moment at breaking and the bending moment of the whole plant. This dual-optimization strategy, which simultaneously enhances stem strength while reducing the gravitational load, represents an ideal ideotype for DDSR breeding. It suggests that selection for lodging resistance should not focus exclusively on either M or WP, but rather on their balanced improvement. Analysis of the bending moment of the whole plant revealed that the length from broken point to panicle top in SLR decreased by 10.9% compared to WLR, while there was no significant difference in the plant part above the broken point among the varieties. Therefore, the lower bending moment of the whole SLR plant can be attributed to the reduction in length from the broken point to the panicle top (Figure 3). The observed increase in bending stress among SLR varieties reflects the enhanced material strength of the culm tissue per unit cross-sectional area, which is fundamentally determined by cell wall composition, particularly lignin and cellulose content, as well as the density and arrangement of vascular bundles [34]. In this study, the bending stress of SLR varieties was 27.9% and 81.9% higher than that of MLR and WLR varieties, respectively (Figure 4), indicating substantially superior tissue quality in lodging-resistant genotypes. This interpretation aligns with the findings of Li et al. [31], who similarly reported that elevated bending stress contributes substantially to improved stem strength in rice. Notably, we observed that the cross-section modulus of MLR varieties was markedly lower than that of WLR varieties, further underscoring the importance of material properties over geometric dimensions. Collectively, these results suggest that in dry direct-seeded systems, selection for improved tissue quality, reflected by higher bending stress, may represent a more efficient strategy for enhancing lodging resistance than merely increasing culm dimensions.

### 3.2. Morphological Differences in Rice Germplasm Under Dry Direct Seeding

The lodging resistance of a crop is also affected by the morphological characteristics of individuals [39,40,41]. Previous studies have reported that the lodging index is closely related to plant height and center-of-gravity height, with tall-stalk varieties tending to have a higher risk of lodging [7,17,42]. In this study, the plant height and center-of-gravity height of SLR varieties were significantly lower than those of MLR and WLR varieties (Figure 5), such that the length from the broken point to the panicle top was also lower (Figure 3). This finding aligns with the well-established biomechanical principle that plant height acts as a lever arm, exponentially increasing the bending moment at the stem base [13].

Our results further revealed that the difference in plant height between SLR and MLR varieties was primarily attributable to variations in the length of basal internodes. Specifically, SLR varieties reduced plant height by shortening the basal internode (Figure 6A,D), a pattern consistent with previous reports demonstrating that plant height can be effectively controlled by reducing basal internode length while increasing the length of the neck–panicle node [20]. Moreover, this study demonstrated that the lodging index was significantly positively correlated with the plant height component index (PHCI) of the basal second internode (Figure 9), and PHCI values differed markedly among variety types, with SLR exhibiting the lowest values (Figure 5C,F). These results indicate that a higher proportion of basal internode length relative to total plant height exacerbates lodging susceptibility.

It is important to note that the relationship between plant height and lodging is not always linear. In the Green Revolution, the introduction of semi-dwarfing genes (*sd1*) dramatically reduced lodging by shortening culm length, yet excessive dwarfing can lead to yield penalties due to reduced canopy photosynthesis and biomass accumulation [36]. Therefore, selectively shortening basal internodes rather than uniformly reducing plant height offers an effective strategy for improving lodging resistance without sacrificing canopy light interception. The PHCI thus emerges as a valuable selection criterion for breeders aiming to decouple lodging resistance from overall plant stature.

The lodging resistance of the stem was also affected by the parameters of the basal internode [43,44]. Many studies have demonstrated that increasing culm diameter substantially improves stem breaking strength, thereby enhancing the lodging resistance of otherwise high-stalk varieties [26,27,28]. However, it was reported that the breaking strength of the stem is not always related to the stem diameter, while the wall thickness of the culm is positively correlated with the stem strength [31]. In this study, outer diameter, inner diameter, and culm wall thickness were found to be significantly positively correlated with the cross-sectional modulus (Figure 9). However, bending stress showed a significant negative correlation with inner diameter and no significant correlation with culm wall thickness. This negative correlation between inner diameter and bending stress can be explained by the trade-off between hollowness and tissue density. A larger inner diameter reduces the amount of load-bearing material per unit cross-sectional area, thereby decreasing the intrinsic material strength (BS) even if the overall section modulus (SM) increases [13,30]. Furthermore, the structural equation model (Figure 10) revealed that the inner diameter exerted both direct and indirect positive effects on the lodging index. Compared with WLR, SLR exhibited a smaller inner diameter in 2020 (Figure 7B). Considering that bending stress is the most critical mechanical parameter influencing the lodging index, increasing culm wall thickness appears to be more effective than increasing inner diameter in balancing the relationship between the cross-sectional modulus and bending stress, thereby enhancing stem strength. In addition, we observed that culm dry weight had both direct and indirect negative effects on the lodging index (Figure 10). SLR also exhibited a higher culm dry weight, while the differences among variety types were not statistically significant (Figure 7B,E). These findings suggested that culm dry weight plays a crucial role in enhancing stem stiffness [30,41]. Culm dry weight is a proxy for biomass accumulation and structural carbohydrate deposition in the basal internode. A higher dry weight per unit length reflects greater cell wall thickening and lignification, which directly contribute to increased bending stress and breaking resistance [34]. Moreover, previous studies [45,46] have reported a significant correlation between breaking strength and leaf sheath density. A higher leaf sheath density was found to have a substantial compensatory effect on culm strength, thereby enhancing stem breaking strength. The leaf sheath acts as an external cylinder providing additional mechanical support to the enclosed culm, particularly during the grain-filling stage when the stem is subjected to increasing gravitational load [32]. In the context of DDSR, where basal internodes are inherently slender, selection for tightly clasping, high-density leaf sheaths may offer a complementary strategy to improve overall stem integrity. Therefore, in addition to increasing culm dry weight, enhancing leaf sheath density appears to be an effective approach to improve stem strength.

### 3.3. Differences in Yield Formation of Rice Germplasm Under Dry Direct Seeding

Improving the lodging resistance of varieties plays a crucial role in both grain yield formation and yield stability. Since the 1960s, the Green Revolution has significantly increased the grain yield and reduced the lodging risk of crops [31,47,48]. However, it is generally believed that excessive plant dwarfing may have a negative effect on grain yield [49,50]. In this study, SLR had a lower plant height compared with MLR and WLR (Figure 5A,D), but there were no significant differences in grain yield among variety types (Table 4). In terms of yield components, SLR exhibited a higher grain-filling rate and grain weight, but a lower number of spikelets per panicle. This pattern of yield compensation, whereby reduced sink size is offset by enhanced sink strength, represents a key physiological mechanism underlying the maintenance of yield potential in lodging-resistant ideotypes. Recent genetic studies have further demonstrated that lodging resistance and yield potential can be synergistically improved through optimized plant architecture. For instance, the *IPA1* gene enhances lodging resistance by increasing culm diameter while simultaneously improving yield through balanced tillering and panicle development [51]. Similarly, the major QTL *qSCM4* was shown to increase stem thickness by 25.3% and stem folding resistance by 20.3%, while also enhancing primary branch number per panicle by 16.7% and grain number per panicle by 14.7%, providing a valuable genetic resource for concurrent improvement of culm strength and sink capacity [52]. The reduced plant height and shorter panicle length in SLR varieties may improve assimilate partitioning to the remaining spikelets by reducing intra-plant competition for carbohydrates during grain filling [53]. Additionally, the enhanced stem strength and structural integrity of SLR varieties likely maintain better canopy light distribution and photosynthetic capacity during the critical grain-filling period, thereby supporting higher grain-filling rates. Previous studies reported that under direct seeding conditions, increasing the number of effective panicles can significantly increase the number of spikelets per unit area, resulting in higher grain yield [54]. In this study, there was no significant difference in panicle weight between SLR and WLR varieties (Figure 8B,E). However, SLR significantly reduced panicle length (Figure 8A,D) and thus plant height and center-of-gravity height. The reduction in panicle length without compromising panicle weight suggests that SLR varieties possess a more compact panicle architecture with a higher grain density. This compact panicle type offers dual benefits for DDSR production, as it lowers the center of gravity and thereby reduces the bending moment, while maintaining yield potential through efficient spikelet filling [52]. Such architectural traits should be prioritized in breeding programs targeting improved lodging resistance for direct seeding systems. These results indicate that selecting for compact panicles with reduced length and high grain density represents an effective strategy for improving both lodging resistance and yield stability in dry direct seeding rice.

## 4. Materials and Methods

### 4.1. Experimental Site

**Figure 11:**
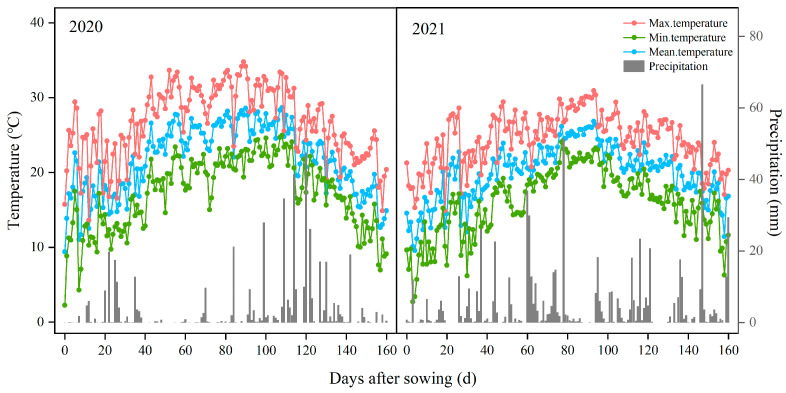
The daily maximum temperatures, mean temperatures, minimum temperatures, and precipitation during the 2020 and 2021 rice growing seasons at Shenyang, Northeast China.

Field experiments were conducted in 2020 and 2021 on a farmer’s field in Shenyang City, Liaoning Province, China (42°24′ N, 122°59′ E). This region has a temperate monsoon climate, with meteorological data obtained from a nearby weather station indicating an annual average temperature of 9.23 °C and average yearly precipitation of 776 mm. The temperature and rainfall during the growing season are shown in Figure 11.

The soil texture was loam, and the plow-layer soil (0–20 cm) had the following properties: organic matter, 14.45 g kg^−1^; total nitrogen, 1.52 g kg^−1^; available nitrogen, 107.42 mg kg^−1^; available phosphorus, 15.84 mg kg^−1^; available potassium, 92.36 mg kg^−1^; pH, 5.77.

### 4.2. Experimental Design and Management

The trial was laid out in a randomized complete block design with three replications. For both years, seventy-nine *japonica* inbred rice varieties were tested. These varieties were selected from 23 different breeding research institutes based on their good yield performance in transplanting systems (Supplementary Table S1). The plot size was 18 m^2^ (9 m × 2 m), with a row spacing of 20 cm. The seeding rate was set at 90 kg ha^−1^ (390 seeds per square meter), and the sowing depth was 2 cm.

Nitrogen (190 kg N ha^−1^) was applied as urea in four stages: 40% at the basal fertilizer stage, 30% at the tillering stage, 20% at the panicle initiation stage, and 10% at the heading stage. Phosphorus (100 kg P_2_O_5_ ha^−1^ as triple superphosphate) was applied one day before sowing. Potassium (75 kg K_2_O ha^−1^ as potassium sulfate) was applied equally in two stages, one day before sowing and at the panicle initiation stage. All treatments were flooded after sowing to maintain the soil moisture level, then wetting–drying alternation irrigation was performed after seedling emergence. A flood water depth of 3–5 cm was maintained after the four-leaf stage. Water was then drained at the maximum tillering stage to reduce unproductive tillers, and rewatering was performed at the panicle initiation stage with a 3–5 cm layer of water until the heading stage. Wetting–drying alternation irrigation was performed during the grain-filling stage, and the water was drained at 10 days before maturity. Other management measures, including weed, disease, and pest control, were intensively controlled to avoid yield loss.

### 4.3. Measurements and Sampling

#### 4.3.1. Lodging-Related Traits

Lodging-related traits were measured at 25 days after heading (BBCH 55; 50% of panicle emerged) in 2020 and 2021. Ten representative main stems were selected in each plot, then the samples were brought to the laboratory for the measurement of related traits. The morphological parameters of individual plants measured included plant height, center-of-gravity height, culm length and the plant height component index of the basal 2nd internode. The breaking moment of the basal 2nd internode was determined. Firstly, the plant roots were excised using a sharp scalpel. Subsequently, the basal second internode was carefully detached from the adjacent internodes by cutting precisely at the nodal junctions, ensuring that the internode segment remained intact for subsequent mechanical testing. Then, the breaking strength of the 2nd internode was measured at 5 cm between two support points with a plant stem strength tester (DIK-7401, Daiki Rika Kogyo Co. Ltd., Tokyo, Japan). After the 2nd internode was pressed until broken, the fresh weight of the plant above the breaking point was recorded immediately to avoid weight loss. Subsequently, the culm diameter and culm wall thickness near the broken position were measured using a digital caliper at the 2nd internode from the ground. Meanwhile, the culm length of the basal 1st and 2nd internodes was also measured to calculate the plant height component index. The dry weight of the culm was measured after oven-drying at 80 °C until constant weight was achieved. The lodging-related parameters were calculated as follows:

Bending moment of the whole plant (WP, g cm) = Length from broken point to panicle tip (SL, cm) × Fresh plant weight of the plant part above the broken point (FW, g)(1)

Bending moment at breaking (M, g cm) = 1/4 × breaking strength (kg) × Distance between fulcra (cm) × 10^3^ (2)

Lodging index (LI, %) = Bending moment of the whole plant/Bending moment at breaking × 100(3)

Cross-section modulus (SM, mm^3^) = π/32 × (a_1_^3^b_1_ − a_2_^3^b_2_)/a_1_ (4)

Bending stress (BS, g mm^−2^) = Bending moment at breaking/cross section modulus (5)

Culm dry matter (CDW, mg cm^−1^) = W_2_/I_2_. (6)

Plant height component index (PHCI, %) = I_2_/(I_1_ + I_2_)× 100 (7)

Culm phenotype index (CPI, %) = the 2nd internode diameter (mm)/ the 2nd internode length (cm) × 100(8)

where a_1_ and a_2_ are the outer and inner diameters of the minor axis in an elliptical cross-section, respectively; b_1_ and b_2_ are the outer and inner diameters of the major axis in an elliptical cross-section, respectively; W_2_ is the weight of the basal 2nd internode; and I_1_ and I_2_ are the culm length of the 1st and 2nd internodes from the ground, respectively.

#### 4.3.2. Grain Yield, Yield Components and Panicle Traits

At maturity, 5 m^2^ (the middle five rows by 5 m) was harvested from each plot to measure the grain yield. The grain yield was adjusted to a moisture content of 14.5% using a moisture detector (PM-8188-A, Kett Electric Laboratory Co., Ltd., Tokyo, Japan). Plants were sampled from a 1m^2^ area to determine the yield components. After the number of panicles m^–2^ was recorded, the samples were threshed by hand and the number of total spikelets was counted. Then, the samples were submerged in tap water to separate filled and unfilled spikelets. After the samples were air-dried, the filled and unfilled spikelets were counted and weighed, and the grain-filling rate and 1000-grain weight were calculated. Furthermore, ten representative main stems were systematically sampled from each experimental plot. The detached panicles were suspended in mesh bags for natural air-drying, followed by quantitative assessments of panicle length, panicle weight, and grain density.

### 4.4. Statistical Analysis

Microsoft Excel 2016 Software (Microsoft Corp., Redmond, WA, USA) was used to organize the data. Analysis of variance (ANOVA), cluster analysis, correlation analysis and principal component analysis were performed using R (R 4.1.2). Differences among treatment means were determined by the Tukey–Kramer method for multiple comparisons tests at the 0.05 probability level. The Spearman’s correlation coefficient was used to analyze the relationship between lodging-related traits at the 0.05 and 0.01 probability levels. Piecewise structural equation modeling (piecewiseSEM) was employed to evaluate the direct and indirect pathways through which plant traits influence variations in the lodging index by affecting mechanical parameters. All observed variables were divided into composite variables first and then included in SEMs. These analyses were conducted using the “piecewiseSEM” package in R. We used Fisher’s C test to judge the goodness of the modeling results. The models were modified stepwise according to the pathway significance (*p* < 0.05) and the goodness of the model (0 ≤ Fisher’s C/df ≤ 2 and 0.05 < *p* ≤ 1.00).

## 5. Conclusions

The lodging index is an important index that can be used to evaluate the lodging risk of crops. In this study, 79 rice varieties were divided into strong lodging resistance (SLR), medium lodging resistance (MLR) and weak lodging resistance (WLR) varieties. SLR varieties achieved a markedly lower lodging index by effectively balancing the bending moment of the whole plant and the bending moment at breaking, the former reduced due to a shorter plant height and a lower center of gravity and the latter enhanced due to elevated bending stress. Structural equation modeling further identified culm dry weight, inner diameter, and culm phenotype index as the primary determinants driving lodging index variation. Importantly, this study demonstrates that strong lodging resistance does not compromise grain yield. Although SLR varieties produced fewer spikelets per panicle and had shorter panicles, these differences were fully offset by significantly higher grain-filling rates and 1000-grain weights, resulting in comparable yields across variety types. These findings provide a practical framework for breeding lodging-resistant, high-yielding *japonica* varieties adapted to dry direct seeding systems in Northeast China.

## Figures and Tables

**Table 1:** Analysis of variance of physical characteristics related to lodging.

Analysis of Variance	LI	WP	M	SL	FW	BS	SM
Year (Y)	NS	*	NS	NS	NS	NS	NS
Variety (V)	*	**	**	**	*	**	**
Y × V	*	*	NS	NS	NS	NS	NS

LI, lodging index; WP, bending moment of the whole plant; M, bending moment at breaking; SL, the length from the broken point to the panicle top; FW, fresh weight of the plant part above the broken point; BS, bending stress; SM, cross-section modulus. NS, not significant at *p* = 0.05; *, *p* < 0.05; **, *p* < 0.01.

**Table 2:** Analysis of variance of morphological traits of individual plants.

Analysis of Variance	PH	CGH	PHCI
Year (Y)	NS	**	NS
Variety (V)	**	**	**
Y × V	NS	**	NS

PH, plant height; CGH, center-of-gravity height; CL, culm length of basal 2nd internode; PHCI, plant height component index of basal 2nd internode. NS, not significant at *p* = 0.05; **, *p* < 0.01.

**Table 3:** Analysis of variance of internode traits.

Analysis of Variance	CL	CDW	CPI	OD	ID	CWT
Year (Y)	NS	**	NS	NS	NS	NS
Variety (V)	*	NS	*	*	*	**
Y × V	NS	*	*	**	NS	NS

CL, culm length of basal 2nd internode; CDW, culm dry weight; CPI, culm phenotype index; OD, outer diameter; ID, inner diameter; CWT, culm wall thickness. NS, not significant at *p* = 0.05; *, *p* < 0.05; **, *p* < 0.01.

**Table 4:** Grain yield and yield components of different varieties in 2020 and 2021.

Year	Variety Type	Grain Yield (t ha^−1^)	Effective Panicles (m^−2^)	Spikelets Per panicle	Spikelets m^−2^ (×10^3^)	Grain-Filling Rate (%)	1000-Grain Weight (g)
2020	SLR	7.37 a	381.81 a	93.71 b	35.06 b	90.03 a	25.39 a
	MLR	7.32 a	382.55 a	100.20 ab	37.69 ab	88.34 ab	24.44 ab
	WLR	7.44 a	391.50 a	108.56 a	41.85 a	84.39 b	23.49 b
2021	SLR	7.43 a	381.67 a	96.67 a	36.30 a	90.38 a	25.50 a
	MLR	7.42 a	380.76 a	103.46 a	38.89 a	88.09 ab	24.45 ab
	WLR	7.46 a	392.00 a	108.64 a	42.13 a	84.24 b	23.45 b
Analysis of variance							
Year (Y)		NS	NS	NS	NS	NS	NS
Variety (V)		NS	NS	*	*	**	**
Y × V		NS	NS	NS	NS	NS	NS

SLR, strong lodging resistance varieties; MLR, medium lodging resistance varieties; WLR, weak lodging resistance varieties. Different lowercase letters within a column indicate significant (*p* < 0.05) differences among varieties. NS, not significant at *p* = 0.05; *, *p* < 0.05; **, *p* < 0.01.

## Data Availability

The data used to support the findings of this study can be made available by the corresponding author upon request.

## Supplementary Materials

The following supporting information can be downloaded at https://www.mdpi.com/article/10.3390/plants15091287/s1: Table S1: The 79 rice varieties collected from Northeast China.

## References

[1] Khush G.S. (2013). Strategies for increasing the yield potential of cereals: Case of rice as an example. Plant Breed..

[2] Deng F., He L.H., Chen D., Zhang C., Tian Q.L., Wu Z.Y., Li Q.P., Zeng Y.L., Zhong X.Y., Chen H. (2022). Growth characteristics and grain yield of machine-transplanted medium indica hybrid rice with high daily yield. J. Integr. Agric..

[3] Sandhu N., Subedi S.R., Yadaw R.B., Chaudhary B., Prasai H., Iftekharuddaula K., Thanak T., Thun V., Battan K.R., Ram M. (2017). Root traits enhancing rice grain yield under alternate wetting and drying condition. Front. Plant Sci..

[4] Liu H., Hussain S., Zheng M., Peng S., Huang J., Cui K., Nie L. (2015). Dry direct-seeded rice as an alternative to transplanted-flooded rice in Central China. Agron. Sustain. Dev..

[5] Namikawa M., Matsunami T., Yabiku T., Takahashi T., Matsunami M., Hasegawa T. (2023). Analysis of yield constraints and seasonal solar radiation and temperature limits for stable cultivation of dry direct-seeded rice in northeastern Japan. Field Crops Res..

[6] Ahmed S., Alam M.J., Hossain A., Islam A.K.M.M., Awan T.H., Soufan W., Qahtan A.A., Okla M.K., El Sabagh A. (2020). Interactive Effect of Weeding Regimes, Rice Cultivars, and Seeding Rates Influence the Rice-Weed Competition Under dry Direct-Seeded Condition. Sustainability.

[7] Farooq M., Siddique K.H.M., Rehman H., Aziz T., Lee D.J., Wahid A. (2011). Rice direct seeding: Experiences, challenges and opportunities. Soil Tillage Res..

[8] Yu S., Lee H., Lo S., Ho T.D. (2021). How does rice cope with too little oxygen during its early life?. New Phytol..

[9] Setter T.L., Laureles E.V., Mazaredo A.M. (1997). Lodging reduces yield of rice by self-shading and reductions in canopy photosynthesis. Field Crops Res..

[10] Kashiwagi T., Sasaki H., Ishimaru K. (2005). Factors Responsible for Decreasing Sturdiness of the Lower Part in Lodging of Rice (*Oryza sativa* L.). Plant Prod. Sci..

[11] Lang Y.Z., Yang X.D., Wang M.E., Zhu Q.S. (2012). ffects of Lodging at Different Filling Stages on Rice Yield and Grain Quality. Rice Sci..

[12] Shah A.N., Tanveer M., Rehman A.U., Anjum S.A., Iqbal J., Ahmad R. (2017). Lodging stress in cereal—effects and management: An overview. Environ. Sci. Pollut. Res..

[13] Berry P.M., Sterling M., Spink J.H., Baker C.J., Sylvester-Bradley R., Mooney S.J., Tams A.R., Ennos A.R. (2004). Understanding and Reducing Lodging in Cereals. Adv. Agron..

[14] Crook M.J., Ennos A.R. (1995). The effect of nitrogen and growth regulators on stem and root characteristics associated with lodging in two cultivars of winter wheat. J. Exp. Bot..

[15] Berry P.M., Spink J.H., Gay A.P., Craigon J. (2003). A comparison of root and stem lodging risks among winter wheat cultivars. J. Agric. Sci..

[16] Kashiwagi T., Togawa E., Hirotsu N., Ishimaru K. (2008). Improvement of lodging resistance with QTLs for stem diameter in rice (*Oryza sativa* L.). Theor. Appl. Genet..

[17] Wang W.X., Du J., Zhou Y.Z., Zeng Y.J., Tan X.M., Pan X.H., Shi Q.H., Wu Z.M., Zeng Y.H. (2021). Effects of different mechanical direct seeding methods on grain yield and lodging resistance of early indica rice in South China. J. Integr. Agric..

[18] Wang W., Peng S., Liu H., Tao Y., Huang J., Cui K., Nie L. (2017). The possibility of replacing puddled transplanted flooded rice with dry seeded rice in central China: A review. Field Crops Res..

[19] Wang X.Y., Xu L., Li X.X., Yang G.D., Wang F., Peng S.B. (2022). Grain yield and lodging-related traits of ultra-short-duration varieties for direct-seeded and double-season rice in Central China. J. Integr. Agric..

[20] Zhang J., Li G., Song Y., Liu Z., Yang C., Tang S., Zheng C., Wang S., Ding Y. (2014). Lodging resistance characteristics of high-yielding rice populations. Field Crops Res..

[21] Kashiwagi T., Hirotsu N., Ujiie K., Ishimaru K. (2010). Lodging resistance locus *prl5* improves physical strength of the lower plant part under different conditions of fertilization in rice (*Oryza sativa* L.). Field Crops Res..

[22] Liu Q., Ma J., Zhao Q., Zhou X. (2018). Physical Traits Related to Rice Lodging Resistance under Different Simplified--Cultivation Methods. Agron. J..

[23] Mulder E.G. (1954). Effect of mineral nutrition on lodging of cereals. Plant Soil.

[24] Luo X., Wu Z., Fu L., Dan Z., Yuan Z., Liang T., Zhu R., Hu Z., Wu X. (2022). Evaluation of lodging resistance in rice based on an optimized parameter from lodging index. Crop Sci..

[25] Zhu G., Li G., Wang D., Yuan S., Wang F. (2016). Changes in the Lodging-Related Traits Along with Rice Genetic Improvement in China. PLoS ONE.

[26] Kashiwagi T., Ishimaru K. (2004). Identification and Functional Analysis of a Locus for Improvement of Lodging Resistance in Rice. Plant Physiol..

[27] Nomura T., Arakawa N., Yamamoto T., Ueda T., Adachi S., Yonemaru J.I., Abe A., Takagi H., Yokoyama T., Ookawa T. (2019). Next generation long-culm rice with superior lodging resistance and high grain yield, Monster Rice 1. PLoS ONE.

[28] Ookawa T., Hobo T., Yano M., Murata K., Ando T., Miura H., Asano K., Ochiai Y., Ikeda M., Nishitani R. (2010). New approach for rice improvement using a pleiotropic QTL gene for lodging resistance and yield. Nat. Commun..

[29] Tu B., Tao Z., Wang S., Zhou L., Zheng L., Zhang C., Li X., Zhang X., Yin J., Zhu X. (2022). Loss of Gn1a/OsCKX2 confers heavy-panicle rice with excellent lodging resistance. J. Integr. Plant Biol..

[30] Islam M.S., Peng S., Visperas R.M., Ereful N., Bhuiya M.S.U., Julfiquar A.W. (2007). Lodging-related morphological traits of hybrid rice in a tropical irrigated ecosystem. Field Crops Res..

[31] Li W.Q., Han M.M., Pang D.W., Chen J., Wang Y.Y., Dong H.H., Chang Y.L., Jin M., Luo Y.L., Li Y. (2022). Characteristics of lodging resistance of high-yield winter wheat as affected by nitrogen rate and irrigation managements. J. Integr. Agric..

[32] Niklas K. (1998). The Mechanical Roles of Clasping Leaf Sheaths: Evidence from *Arundinaria técta* (Poaceae) Shoots Subjected to Bending and Twisting Forces. Ann. Bot..

[33] Li L., He L., Li Y., Wang Y., Ashraf U., Hamoud Y.A., Hu X., Wu T., Tang X., Pan S. (2023). Deep fertilization combined with straw incorporation improved rice lodging resistance and soil properties of paddy fields. Eur. J. Agron..

[34] Liu S., Huang Y., Xu H., Zhao M., Xu Q., Li F. (2018). Genetic enhancement of lodging resistance in rice due to the key cell wall polymer lignin, which affects stem characteristics. Breed. Sci..

[35] Rani Sinniah U., Wahyuni S., Syahputra B.S.A., Gantait S. (2012). A potential retardant for lodging resistance in direct seeded rice (*Oryza sativa* L.). Can. J. Plant Sci..

[36] Ookawa T., Ishihara K. (1992). Varietal Difference of Physical Characteristics of the Culm Related to Lodging Resistance in Paddy Rice. Jpn. J. Crop Sci..

[37] Quang Duy P., Abe A., Hirano M., Sagawa S., Kuroda E. (2004). Analysis of Lodging-Resistant Characteristics of Different Rice Genotypes Grown Under the Standard and Nitrogen-Free Basal Dressing Accompanied with Sparse Planting Density Practices. Plant Prod. Sci..

[38] Zhao X., Zhou N., Lai S., Frei M., Wang Y., Yang L. (2019). Elevated CO_2_ improves lodging resistance of rice by changing physico-chemical properties of the basal internodes. Sci. Total Environ..

[39] Murai M., Komazaki T., Sato S. (2004). Effects of sd1 and Ur1 (Undulate rachis -1) on Lodging Resistance and Related Traits in Rice. Breed. Sci..

[40] Pan J., Zhao J., Liu Y., Huang N., Tian K., Shah F., Liang K., Zhong X., Liu B. (2019). Optimized nitrogen management enhances lodging resistance of rice and its morpho-anatomical, mechanical, and molecular mechanisms. Sci. Rep..

[41] Liao P., Bell S.M., Chen L., Huang S., Wang H., Miao J., Qi Y., Sun Y., Liao B., Zeng Y. (2023). Improving rice grain yield and reducing lodging risk simultaneously: A meta-analysis. Eur. J. Agron..

[42] Wu M., Jiang H., Wei Z., Li W., Gao K., Wang D., Wei X., Tian P., Cui J., Di Y. (2023). Influence of Nitrogen Application Rate on Stem Lodging Resistance Rice Under Dry Cultivation. Agronomy.

[43] Ding C., Luo X.K., Wu Q., Lu B., Ding Y.F., Song J., Li G.H. (2021). Compact plant type rice has higher lodging and N resistance under machine transplanting. J. Integr. Agric..

[44] Hirano K., Okuno A., Hobo T., Ordonio R., Shinozaki Y., Asano K., Kitano H., Matsuoka M. (2014). Utilization of Stiff Culm Trait of Rice smos1 Mutant for Increased Lodging Resistance. PLoS ONE.

[45] Peng D., Chen X., Yin Y., Lu B., Yang W., Tang Y., Wang Z. (2014). Lodging resistance of winter wheat (*Triticum aestivum* L.): Lignin accumulation and its related enzyme activities due to the application of paclobutrazol or gibberellic acid. Field Crops Res..

[46] Wu W., Ma B.L. (2019). Erect-leaf posture promotes lodging resistance in oat plants under high plant population. Eur. J. Agron..

[47] Acreche M.M., Slafer G.A. (2011). Lodging yield penalties as affected by breeding in Mediterranean wheats. Field Crops Res..

[48] Gent M.P.N. (1995). Canopy Light Interception, Gas Exchange, and Biomass in Reduced Height Isolines of Winter Wheat. Crop Sci..

[49] Peng S., Cassman K.G., Virmani S.S., Sheehy J., Khush G.S. (1999). Yield Potential Trends of Tropical Rice since the Release of IR8 and the Challenge of Increasing Rice Yield Potential. Crop Sci..

[50] Xu L., Zhan X., Yu T., Nie L., Huang J., Cui K., Wang F., Li Y., Peng S. (2018). Yield performance of direct-seeded, double-season rice using varieties with short growth durations in central China. Field Crops Res..

[51] Zhang L., Yu H., Ma B., Liu G., Wang J., Wang J., Gao R., Li J., Liu J., Xu J. (2017). A natural tandem array alleviates epigenetic repression of IPA1 and leads to superior yielding rice. Nat. Commun..

[52] Yang X., Lai Y., Wang L., Zhao M., Wang J., Li M., Chi L., Lv G., Liu Y., Cui Z. (2023). Isolation of a novel QTL, qSCM4, associated with strong culm affects lodging resistance and panicle branch number in rice. Int. J. Mol. Sci..

[53] Hu X., Liu Y., Zhong X., Hu R., Li M., Peng B., Pan J., Liang K., Fu Y., Huang N. (2024). Optimized nitrogen management improves grain yield of rice by regulating panicle architecture in south China. Heliyon.

[54] Liang C., Li Y., Zhang K., Wu Z., Liu J., Liu J., Zhou C., Wang S., Li F., Sui G. (2023). Selection and Yield Formation Characteristics of Dry Direct Seeding Rice in Northeast China. Plants.

