# Supplementary material for: Dissecting the Phenotypic Regulation Characteristics of Lodging Resistance in Dry Direct Seeding Rice: Insights from Stem Mechanics and Structural Traits
Source: Plants (Basel). 2026 Apr 22;15(9):1287. doi: 10.3390/plants15091287 (PMC13165418; doi:10.3390/plants15091287)
Supplement: Supplementary file 1 [file plants-15-01287-s001.zip › Supplementary Table S1.pdf]

Table S1. 79 rice varieties collected from Northeast China

No.	Cultivar	Type	Source
1	Shendao334	WLR	Shenyang Agricultural University
2	Jinongda538	WLR	Jilin Agricultural University
3	Jinongda823	MLR	Jilin Agricultural University
4	Tonggeng797	WLR	Tonghua Academy of Agricultural Sciences
5	Shendao333	MLR	Shenyang Agricultural University
6	Shendao702	WLR	Shenyang Agricultural University
7	Shendao14	MLR	Shenyang Agricultural University
8	Shendao7	SLR	Shenyang Agricultural University
9	Shennong315	MLR	Shenyang Agricultural University
10	Shendao72	SLR	Shenyang Agricultural University
11	Tongyu256	MLR	Tonghua Academy of Agricultural Sciences
12	Tongyu838	SLR	Tonghua Academy of Agricultural Sciences
13	Tonghe885	WLR	Tonghua Academy of Agricultural Sciences
14	Xingchen899	WLR	Jilin Xingchen seed Co., Ltd.
15	Jiyang108	SLR	Jiyang Agricultural Science Research Institute
16	Jiyang100	MLR	Jiyang Agricultural Science Research Institute
17	Tongke29	MLR	Tonghua Academy of Agricultural Sciences
18	Hangeng8	SLR	Jilin West Oasis Investment Co., Ltd.
19	Tonggeng890	SLR	Tonghua Academy of Agricultural Sciences
20	Tonghe832	SLR	Tonghua Academy of Agricultural Sciences
21	Shendao47	MLR	Shenyang Agricultural University
22	Beigeng2	SLR	Shenyang Agricultural University
23	Qiuguang	MLR	Jilin Fengyou Agricultural Research Institute
24	Shendao11	MLR	Shenyang Agricultural University
25	Tiegeng15	MLR	Tieling Academy of Agricultural Sciences
26	Shendao240	MLR	Shenyang Agricultural University
27	Shendao215	SLR	Shenyang Agricultural University
28	Shendao88	SLR	Shenyang Agricultural University
29	Jinongda738	WLR	Jilin Agricultural University
30	Jida818	SLR	College of Plant Science, Jilin University
31	Jida898	MLR	College of Plant Science, Jilin University
32	Tongxi935	SLR	Tonghua Academy of Agricultural Sciences
33	Tongke37	MLR	Tonghua Academy of Agricultural Sciences
34	Songliao186	MLR	Songliao Agricultural Science Research Institute
35	Jida618	WLR	College of Plant Science, Jilin University
36	Songgeng16	MLR	Wuchang Rice Research Institute of Heilongjiang Academy of Agricultural Sciences
37	Shendao18	MLR	Shenyang Agricultural University
38	Shendao526	MLR	Shenyang Agricultural University
39	Jigeng49	SLR	Jilin Academy of Agricultural Sciences
40	Shendao536	MLR	Shenyang Agricultural University
41	Shendao49	MLR	Shenyang Agricultural University

42	Shendao69	MLR	Shenyang Agricultural University
43	Shennongdao546	SLR	Shenyang Agricultural University
44	Tonggeng666	SLR	Tonghua Academy of Agricultural Sciences
45	Jihong9	MLR	Jilin Hongye seed Co., Ltd.
46	Songgeng29	MLR	Wuchang Rice Research Institute of Heilongjiang Academy of Agricultural Sciences
47	Yangeng27	MLR	Yanbian Academy of Agricultural Sciences
48	Shendao529	SLR	Shenyang Agricultural University
49	Shendao316	MLR	Shenyang Agricultural University
50	Tongxi937	MLR	Tonghua Academy of Agricultural Sciences
51	Shendao223	MLR	Shenyang Agricultural University
52	Shendao523	MLR	Shenyang Agricultural University
53	Shendao520	SLR	Shenyang Agricultural University
54	Shendao528	MLR	Shenyang Agricultural University
55	Shendao357	WLR	Shenyang Agricultural University
56	Jinongda859	MLR	Jilin Agricultural University
57	Shendao531	MLR	Shenyang Agricultural University
58	Shendao534	MLR	Shenyang Agricultural University
59	Fuyu333	WLR	Jilin West Oasis Investment Co., Ltd.
60	Kaigeng2	MLR	Kaiyuan Agricultural Science Research Institute
61	Zhongke804	MLR	Institute of Genetics and Developmental Biology, Chinese Academy of Sciences
62	Fengmin2000	SLR	Liaoning Fengmin Agricultural High-tech Co., Ltd.
63	Jigeng836	MLR	Jilin Academy of Agricultural Sciences
64	Futian2100	MLR	Shenyang Futian seed Technology Co., Ltd.
65	Fuxingdao39	MLR	Kaiyuan Haoshoucheng crop research institute
66	Zhongyan15	MLR	Jilin Zhongyan Agricultural Development Co., Ltd.
67	Fuxing2838	WLR	Kaiyuan Haoshoucheng crop research institute
68	Fuxing90	MLR	Kaiyuan Haoshoucheng crop research institute
69	Suiyan6	MLR	Heilongjiang Suiyan seed Industry Co., Ltd.
70	Tonghe837	WLR	Tonghua Academy of Agricultural Sciences
71	Tongxi945	MLR	Tonghua Academy of Agricultural Sciences
72	Tongyuan568	MLR	Tonghua Academy of Agricultural Sciences
73	Jiudao48	MLR	Rice Research Institute of Jilin Academy of Agricultural Sciences
74	Tiegeng4	MLR	Tieling Academy of Agricultural Sciences
75	Qinglin598	MLR	Jilin Fengyou Agricultural Research Institute
76	Tiegeng11	MLR	Tieling Academy of Agricultural Sciences
77	Jihong6	WLR	Jilin Hongye seed Co., Ltd.
78	Jigeng528	WLR	Jilin Academy of Agricultural Sciences
79	Songgeng22	WLR	Wuchang Rice Research Institute of Heilongjiang Academy of Agricultural Sciences
